# Persistent Impact of Prior Experience on Spatial Learning

**DOI:** 10.1523/ENEURO.0266-24.2024

**Published:** 2024-09-20

**Authors:** Michelle P. Awh, Kenneth W. Latimer, Nan Zhou, Zachary M. Leveroni, Anna G. Poon, Zoe M. Stephens, Jai Y. Yu

**Affiliations:** ^1^Neuroscience Institute, University of Chicago, Chicago, Illinois 60637; ^2^Department of Neurobiology, University of Chicago, Chicago, Illinois 60637; ^3^Data Science Institute, University of Chicago, Chicago, Illinois 60637; ^4^Grossman Center for Quantitative Biology and Human Behavior, University of Chicago, Chicago, Illinois 60637; ^5^Department of Psychology, University of Chicago, Chicago, Illinois 60637; ^6^Institute for Mind and Biology, University of Chicago, Chicago, Illinois 60637; ^7^University of Chicago Laboratory Schools, Chicago, Illinois 60637

**Keywords:** behavior, generalization, learning, spatial navigation, strategy, transfer

## Abstract

Learning to solve a new problem involves identifying the operating rules, which can be accelerated if known rules generalize in the new context. We ask how prior experience affects learning a new rule that is distinct from known rules. We examined how rats learned a new spatial navigation task after having previously learned tasks with different navigation rules. The new task differed from the previous tasks in spatial layout and navigation rule. We found that experience history did not impact overall performance. However, by examining navigation choice sequences in the new task, we found experience-dependent differences in exploration patterns during early stages of learning, as well as differences in the types of errors made during stable performance. The differences were consistent with the animals adopting experience-dependent memory strategies to discover and implement the new rule. Our results indicate prior experience shapes the strategies for solving novel problems, and the impact of prior experience remains persistent.

## Significance Statement

Prior experience can be useful for solving new problems, especially when learned rules can generalize to new settings. However, it is unclear how prior experience impacts the strategies for learning new rules. We show that prior experience changes the strategies used to solve new problems without changing performance outcomes. The effects of prior experience remained after learning, when performance has plateaued. Our results suggest prior experience has a persistent impact on future problem-solving and may determine how distinct neural processes are engaged to solve new problems.

## Introduction

Individuals learn from distinct and diverse experiences to build general knowledge, which can be applied to new problems (Alonso et al., 2020). New problems can be solved faster when aspects of previous experience, such as learned rules, can be directly applied (E. L. Thorndike and Woodworth, 1901a; Harlow, 1949). When the new problem is distinct from previous experience, impacts on behavior are less consistent (E. L. Thorndike and Woodworth, 1901b; Wiltbank, 1919), making the effect of prior experience challenging to understand.

At the cognitive level, evidence for experience-specific influences on future learning came from studies in the early twentieth century that showed rats, non-human primates, and humans could solve new problems faster after previously encountering related but not identical problems (E. L. Thorndike and Woodworth, 1901a,b,c; Wiltbank, 1919; Ho, 1928; R. L. Thorndike, 1935; Harlow, 1949). A proposed explanation for this effect is “transfer” of learning, where aspects of prior experience are carried over to facilitate performing new tasks. Transfer is typically quantified by differences in performance metrics, such as the number of attempts to reach criterion, the number of errors made, or the time needed to solve a task. A range of transfer outcomes has been observed, ranging from positive transfer (improvement in performance) to neutral effect (no change in performance) to negative transfer (worsening performance; Webb, 1917; Wiltbank, 1919; Dennis et al., 1932). Transfer was reported for different degrees of experience: rats performed fewer errors when learning a new maze even with only partial training of a different maze (Ho, 1928; Bunch and Lang, 1939) or after training in multiple different mazes (Dashiell, 1920; Rashid et al., 2017). Furthermore, prior experience can improve rats’ abilities to solve new problems even when that experience is dissimilar from the new task, such as prior operant learning improving new spatial learning (Adams, 2003) or positive transfer between mazes with different rules (Gallup and Diamond, 1960).

Many early experiments report inconsistent or a lack of performance gains across multiple tasks (Wiltbank, 1919; Dennis et al., 1932). On the one hand, this could indicate that transfer may not apply to some tasks. Alternatively, the metrics used to quantify performance, such as speed or accuracy, may have failed to capture aspects of behavior that did differ. This is especially relevant to exploration in structured environments since humans and non-human animals show intricate exploratory patterns (Uster et al., 1976; Alonso et al., 2021; Rosenberg et al., 2021; Brunec et al., 2023). Moreover, it is possible that for certain tasks, distinct exploration strategies may yield nearly identical learning rates or performance outcomes. Thus, examining detailed aspects of behavioral choices in addition to traditional performance metrics can provide important insights on how prior experience shapes future behavior.

Here, we investigate how prior experience affects the way animals learn and perform a new task. We compare learning in a spatial alternation task between groups of rats that had distinct training histories, having previously learned different spatial alternation tasks with distinct rules. We did not find experience-dependent differences in performance as measured by reward rate. We did find experience-dependent differences in spatial exploration during early learning. Furthermore, we found the types of errors made during stable performance depended on prior experience. We hypothesize these exploration and error patterns are related to the distinct memory requirements from prior experience. Our results demonstrate that experience history can shape problem-solving strategies and the effects of experience can persist throughout learning and performing new tasks. This paves the way for further investigation into how experience can influence the engagement of distinct neural circuits to support future learning.

## Materials and Methods

### Experimental design and animal training

A total of 19 male Long–Evans rats (14–16 weeks old, 475–525 g, Charles River Laboratories) were used in this study. All procedures were performed under approval by the Institutional Animal Care and Use Committee at the University of Chicago, according to the guidelines of the Association for Assessment and Accreditation of Laboratory Animal Care. Animals were kept in a temperature (21°C)- and humidity (50%)-controlled colony room on a 12/12 h light/dark cycle (lights were on from 8:00 to 20:00). Experiments were performed during the light period.

Animals were handled over 4 weeks for habituation to human interaction. Animals were familiarized to foraging for evaporated milk (Carnation) with 5% added sucrose in an elevated black open field box (H, 31 cm; W, 61 cm; L, 61 cm), 10 min per day for 3 d. Reward was randomly dropped inside the open box to encourage foraging. Animals were then food restricted to 90% of their baseline weight for 3 d and trained to run back and forth on an elevated linear track (H, 76 cm; W, 8 cm; L, 60 cm) to consume reward from the ends of the track. Animals were trained for 10 min per day until a performance criterion of 20 rewards per session (for 4–8 d).

Behavior training was conducted on custom-built mazes with interconnecting acrylic track sections (8 cm in width) elevated 76 cm from the floor. Animals were placed on the maze at the start of the session and collected from the maze at the end of the session. The task was automated without experimenter intervention during the session. Reward was delivered by a syringe pump (100 μl at 20 ml/min, NE-500, New Era Pump Systems). Behavior data were recorded using the SpikeGadgets data acquisition system (SpikeGadgets). Our experiment was split into two phases. Rats first were exposed to a differential experience phase and then to a common experience phase. The common experience phase started 2 d after the end of the differential experience phase.

**Figure 1.**
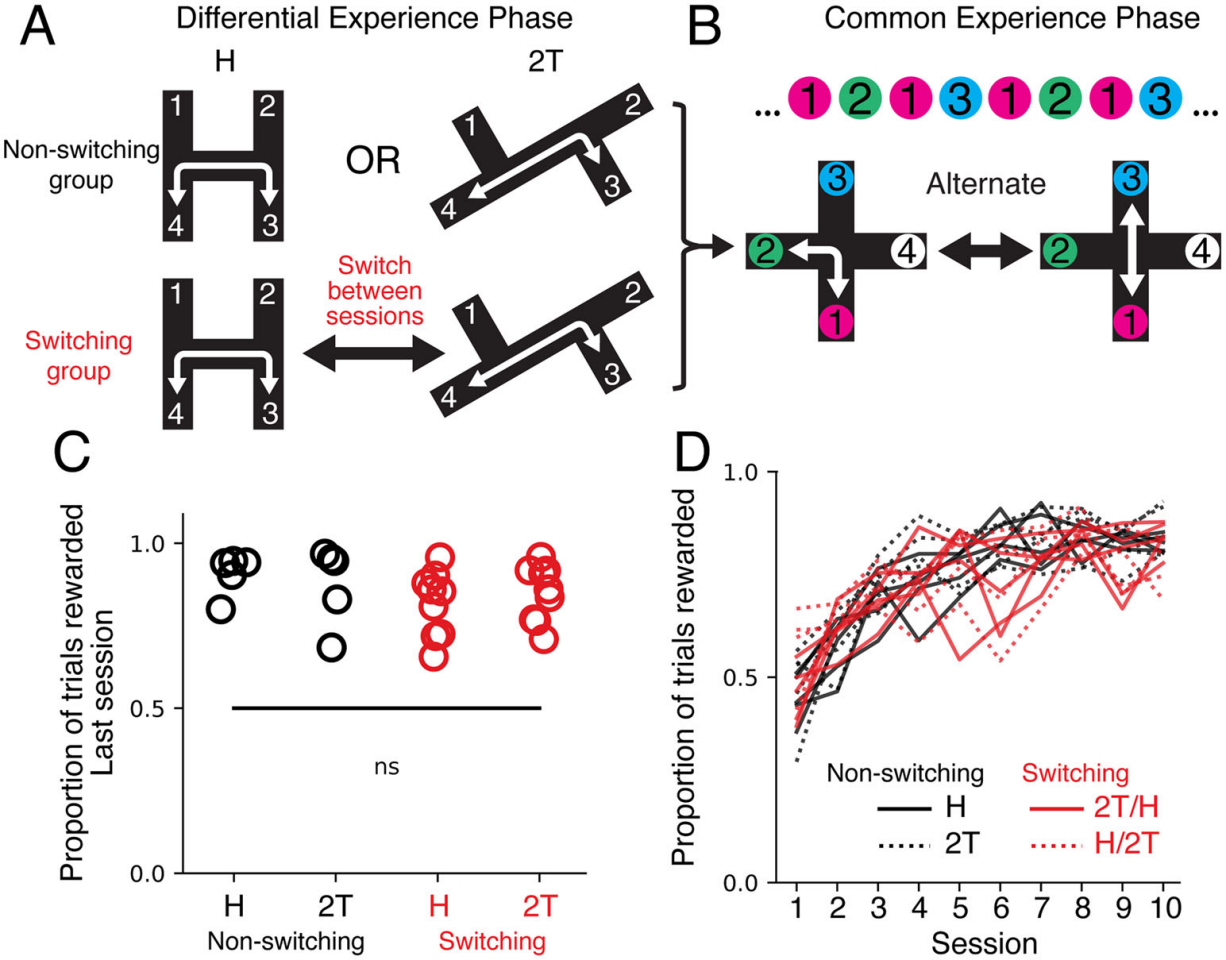
Rats with switching or non-switching experience had similar performance in a novel task. ***A***, Experiment schematic for the differential experience phase. In this phase, all rats were trained for two sessions per day for up to 10 d. Non-switching group rats (H and 2T) learned a single task, either the H maze or the 2T maze, and they trained on the same maze during each training session, twice per day. Switching group (H/2T and 2T/H) rats learned both alternation tasks, H and 2T, counterbalanced for the order of maze sessions. Both the H and 2T mazes have four arm ends; two arm ends are reward locations (green circles). Visits to the other maze ends (white circles) are not rewarded. The rewarded visit sequence is shown. ***B***, Experiment schematic for the common experience phase. All groups of rats learned to navigate the same plus maze task for two sessions per day. The task rule is an alternation sequence between Location 1 and Locations 2 or 3. The rewarded visit sequence is shown. ***C***, The reward rate on the final session of differential experience phase. A two-way ANOVA did not show a significant effect on final performance from experience (*p* = 0.13) or task (*p* = 0.79) or their interaction (*F*_(1, 1) _= 0.70; *p* = 0.41). ***D***, Proportion of trials rewarded per session on the plus maze grouped by experience and task in Phase 1. Pairwise Wilcoxon rank-sum test with Benjamini/Hochberg false discovery rate correction did not show a significant difference between experience (*p* > 0.44 for all pairs) or task (*p* > 0.83 for all pairs) across sessions.

For the differential experience phase, animals were randomly assigned to the non-switching (*n* = 10) and switching group (*n* = 9). An H maze (H) and a double T maze (2T) were used for the differential experience phase training (Fig. 1*A*). Animals obtained rewards from the ends of the maze only when visiting two of the four ends in alternation. Animals performed two 10 min training sessions separated by 3 h, during which the rats were returned to their cages. Training continued until performance exceeded 80% or up to 10 d. The switching group trained on two mazes per day, while the non-switching group trained on one maze twice per day. For the switching group, we controlled the order the rats learned the tasks across each day by assigning 5 rats first to the 2T maze and then to the H maze. The remaining four rats learned the tasks in reverse order.

After the differential experience phase, all animals (*n* = 19) were trained on a plus maze with a different rule (Fig. 1*B*). Both groups were trained to visit three of the four locations in a specific sequence to receive reward at those three locations (Fig. 1*B*). The sequence involved alternating visits between Locations 2 and 3 via Location 1 (1, 2, 1, 3, 1, 2, 1, 3…). This task has the same rule as previous spatial alternation tasks (Frank et al., 2000; Kim and Frank, 2009; Jadhav et al., 2012), the difference being the addition of a fourth arm that is never rewarded. For example, if the rat was at Location 1, it would be rewarded next at Location 2 only if it had previously visited Location 3. Similarly, if the rat was at Location 1, it would be rewarded next at Location 3 only if it had previously visited Location 2. If the rat was at Locations 2 or 3, it would be rewarded only by going to Location 1. This rule ensured the rat received some reward even if it did not completely perform the entire four-visit sequence. The reward sequence involved only visits to Locations 1, 2, and 3 and does not involve any visits to Location 4. Location 4 was not a part of the control logic and is never rewarded.

Animals underwent two 10 min training sessions per day for 5 d. The rats were returned to their cages for 3 h between the two sessions. For each session, the control program was initiated at a random step in the visit location sequence. This was to ensure the animals did not develop a stereotyped action pattern at the start of each session but must correctly alternate in the desired sequence in each session. Since the animal was placed in the center of the maze at the start of each session, it could choose freely which arm to enter first.

### Data processing and statistical analysis

We registered reward location visits based on sensor trigger events and reward delivery based on pump trigger events. All statistical analyses were performed in Python using Numpy, Scipy and Scikit-learn. Nonparametric tests with two-tailed statistics were used. Multiple pairwise-comparison *p* values were corrected for false discovery rate using the Benjamini/Hochberg method.

### Behavior pattern classification

We started with a sequence of reward location visits, which represent first-order patterns. We converted this sequence into second-order behavior patterns given each pair of transitions requires one to two movement choices: left turn (L), straight (S), or right turn (R). We then classified third-order patterns as the transition between second-order actions, such as repeating turns (LL or RR), repeating straight (SS), transitioning between left or right turns (LR, RL), or between turns and straight (RS, LS, SR, SL).

### Action sequence probabilities

We calculated the probability of observing a specific action sequence for all possible three-trial sequences, for example (L, LL, LLL, …). Data for all rats was in the form of a *m* × *n* matrix, with *m* being each animal and *n* being all the possible sequences. To visualize the probability matrix as a dendrogram, we used the Python networkx package (https://networkx.org/). To visualize the similarity between the probability matrices for the non-switching and switching groups, we used principal component analysis (PCA) to reduce the dimensionality of this matrix. To quantify similarity, we calculated the pairwise cosine similarity for a pair of animals across all principal components. This was done for within (switching to switching, non-switching to non-switching) and across (switching to non-switching) group comparisons.

### Modified distance-dependent Chinese restaurant process model

We aimed to summarize statistically how the actions of each rat in the plus maze depended on the recent trials and how the distribution of choices changed over the course of learning. Given the sequence of trials performed by an animal, we modeled the action on a trial as a probability distribution that depended on the past trial and the number of trials performed. The dependency on the number of trials allowed the model to account for the changes in the animals’ behavior during learning. This contrasts with a typical Markov model, which assumes behavior has a fixed dependency on the immediate past trials but not the history of trials performed.

To accomplish this, we modeled the sequence of actions (left, right, or straight) performed by each rat using a sequential distance-dependent Chinese restaurant process (ddCRP) model (Blei and Frazier, 2011). We modified the model by adding a parameter that specifically controls the contribution of the last trial to the upcoming choice. The ddCRP defines a generative stochastic process in which the probability of the action on the *i*th trial depends on the outcomes of the previous trials. The probability of observing action A on trial i $\left(y_{i}\right)$ is given as follows:

$$p(y_{i}=A\;|y_{1:i-1},\theta )\propto \alpha G_{i}(A)+\overset{i-1}{\underset{\;j=1\;i,y_{j}=A}{\sum }}\;f(i,j),$$

where the distance function between trials i and j is as follows:

$$f(i,j)\;=\;\exp\;\left(-\frac{\left|i-j\right|}{\tau }\right)\overset{2}{\underset{d=1}{\prod }}\;(1-C_{d}{)}^{m_{d}(i,j)},$$

where $m_{d}(i,j)=0$ if trials i and j share the same context of depth d: that is, the sequence of d actions immediately preceding trials i and j are the same. Otherwise, we set $m_{d}(i,j)=1$. The timescale parameter of the distance function, τ>0, determines how predictive actions from the past are of the current trial. Low values of τ indicate that the actions at the beginning of the session are not informative of the animals’ behavior at the end of the session. This timescale gives the process the “distance-dependent” property in comparison with the standard Chinese restaurant process, which weighs all previous observations with Weight 1. The context parameters, $C_{d}\;\in \;[0,1]$, determine how much choice depends on specific actions permed on the *d* previous trials (the context). If $C_{d}\;=\;1$, then context is weighted heavily by the model: the actions performed in one context do not inform the actions in a different context. If $C_{d}\;=\;0$, the context is not predictive of the actions.

The remaining two parameters define the base measure, $G_{i}$: the prior probability over the actions as follows:

$$G_{i}(A)\propto \beta \;\mathrm{if}\;y_{i-1}=A\;\mathrm{and}\;G_{i}(A)\propto \;1\;\mathrm{if}\;y_{i-1}\neq A.$$

The concentration parameter, α>0, determines bias for selecting the choice on each trial from the base distribution. The bias parameter, β>0, is included to alter the base distribution. The value of this parameter accounts for how a fixed switch-stay bias could account for the animals’ sequence of actions. Actions are drawn from the uniform distribution a priori if β=1. For β<1, actions are less likely to be repeated, and for β>1, choices are more likely to be repeated.

Our approach extended the ddCRP model for sequences to include recent context within the distance function. This approach was inspired by models that use hierarchical Dirichlet priors to regularize estimation of Markov models (Wood et al., 2009). However, our method took advantage of the fact that the distance function already weighs the previous observations differently. Thus, we could incorporate dependencies on recent actions without a more complex hierarchical model in contrast to a recently proposed statistical model of behavioral sequences (Éltető et al., 2022).

We fitted the model in a Bayesian framework using Markov chain Monte Carlo (MCMC) methods implemented using the Stan modeling platform (STAN Development Team, 2023). Convergence of the MCMC procedure was assessed using the $\hat{R}$ metric (Vehtari et al., 2021) with four independent chains of 1,000 samples each. We used the posterior median as a point estimate for individual parameters. The prior distributions for the parameters were independent for each parameter as follows:

τ∼Gamma(2,20),

$$C_{d}∼\mathrm{Uniform}(0,1),$$

α∼Gamma(2,2),

β∼Gamma(20,1/20),

where the gamma distributions were parameterized as shape and scale.

### Markov models

We constructed a simulation based on the sequences of actions during the first phase of the experiment to test how a strategy based on the learned H/2T mazes, combined with random exploration, would compare with the animals’ performance while learning the plus maze. We characterized each rat's behavior on the last 5 d of the first phase as a second-order Markov chain. The Markov chain defined the probability of turning to the left, right, or going straight at intersections on the current trial given the previous two trials. The individual actions needed to travel between two reward locations were based on the geometric structure of each maze. We estimated the transition probabilities based on the counts of the observed sequences:

$$P(y_{t}=c|y_{t-1}=b,y_{t-2}=a)\propto N_{\mathrm{abc}}+1,$$

for a,b,c∈ {left, right, straight} where $N_{abc}$ was the number of times the animal performed the sequence abc. The plus one regularized the estimate and ensured that all probabilities were positive.

The switching group ran two mazes on each day (one maze in the morning and the other in the afternoon). We therefore computed two transition probabilities for each rat: one using the morning sessions, $P_{M}$, and one using the afternoon sessions, $P_{A}$. We also defined an exploratory/flat transition probability, $P_{E}$, where the probability of performing any of the three actions (left, straight, or right) was one-third regardless of recent history.

We then modeled the animal's performance on the cross maze as a combination of transition probabilities. The animal's actions at each time were governed by a behavioral state corresponding to the morning, afternoon, or exploratory transition probabilities ( M, A, and E, respectively). On each trial, the behavioral state was updated, and then an action was selected based on the past two actions given the current state. We parameterized the transitions between behavioral states using two parameters: the probability of exploration, $q_{E}$, and the probability of switching task models, $q_{S}$. The probability of going from state M or state A to state E was $q_{E}$. The probability of going from state M to state A (or vice versa) was $q_{S}(1-q_{E})$. The probability of going from state E to state M (or A) was $\frac{1}{2}q_{E}$.

Combining these elements, we constructed a Markov chain with a state defined by three variables: the behavioral state and the previous two actions where the probability of transitioning $\left(s_{t-1},y_{t-1},y_{t-2})\to (s_{t},y_{t},y_{t-1}\right)$ was the product of the probability of transitioning from behavioral state $s_{t-1}$ to $s_{t}$ times $P_{s_{t}}(y_{t}|y_{t-1},y_{t-2})$. Using standard Markov chain analysis, we computed the steady-state distribution $\pi (s_{t},y_{t},y_{t-1})$. We could then compute the probability of, for example, turning after going straight (str.) as follows:

$$p(\mathrm{turn}\;\mathrm{after}\;\mathrm{str}.)=\underset{s_{t},s_{t-1}\in A,M,E,y_{t}\in \{\mathrm{left},\mathrm{right}\},y_{t-2}\in \{\mathrm{left},\mathrm{right},\mathrm{straight}\}}{\sum }\;P_{s_{t}}(y_{t}|y_{t-1}=\mathrm{str}.,\;y_{t-2})P(s_{t}|s_{t-1})\frac{\pi (s_{t-1},y_{t-1}=\mathrm{str}.,y_{t-2})}{\pi (y_{t-1}=\mathrm{str}.)},$$

$$\pi (y_{t-1}=\mathrm{str}.)=\underset{s_{t-1}\in A,M,E,y_{t-2}\in \{\mathrm{left},\mathrm{right},\mathrm{str}.\}}{\sum }\;\pi (s_{t-1},y_{t-1}=\mathrm{str}.,\;y_{t-2}).$$

### Code accessibility

The code for ddCRP is available at https://github.com/latimerk/hddCRP. The computations were performed on a Linux-based system.

10.1523/ENEURO.0266-24.2024.d1 Extended Data 1 Code for Chinese Restaurant Process model. Download Extended Data 1, ZIP file.

### Error analysis

The alternation rule for the plus maze involved repeating a sequence of reward location visits (1, 2, 1, 3, …). Errors performing this sequence could be classified into two types. Alternation errors occurred when the animal was at Location 1 and failed to alternate, instead returning to a previously visited location. The two types of alternation errors were 2 − 1→{2 or 4} when 2 − 1→3 was correct or 3 − 1→{3 or 4} when 3→1→2 was correct. Reference location errors occurred when the animal was at Locations 2 or 3 and failed to return to Location 1. The two reference location errors were 1 − 2→{3 or 4} when 1 − 2→1 was correct or 1 − 3→{2 or 4} when 1 − 3→1. We defined a lapse as an error following a complete sequence of four correct visits. This selected for sudden lapses given the animal had knowledge of the task rule.

Next, we constructed models in which the animal's behavior was perfect on most trials, but on *p*_lapse_ trials, the behavior followed a “lapse” strategy. The first lapse strategy was defined based on turn preferences relative to the current location rather than by specific location (Fig. 7, Extended Data Fig. 7-1). We allowed for the possibility that the animal was accessing turn preferences for the plus, H or 2T mazes, since the same actions (left, straight and right) applied in all mazes. Each task had a specific probability for each action. If the preference was defined by the plus maze task, the turns on lapse trials were made with probability *P*(left = 0.25), *P*(straight = 0.5), and *P*(right = 0.25) because there were twice as many straight choices for each turn choice on correct sequences (L, R, S, S). Selecting based on the preference, we again computed the probability of the alternation errors on lapse trials: *P*(choice ≠ 2 | lapse at 3−1) = 3/4 and *P*(choice ≠ 3 | lapse at 2−1) = 1/2 and the total alternation error probability became *P*(out error | making out decision and lapse) = 5/8. The reference location error probabilities on lapse trials were *P*(choice ≠ 1 | lapse at 1−2) = 3/4 and *P*(choice ≠ 1 | lapse at 1−3) = 1/2 and the total reference location error rate on lapse trials was *P*(in error | making in decision and lapse) = 5/8. Under this turn-based strategy, the probability of an alternation error was equal to a reference location error assuming lapses were equally likely on both decision types. This equality was due to the balanced construction of the task: there was one turn for departing or returning to Location 1, one straight choice departing or returning to Location 1, and the probability of turning left equaled the probability of turning right.

Similarly, the ideal turn probabilities for the 2T task were *P*(left = 1/4), *P*(straight = 1/2), and *P*(right = 1/4): going for 3–4 required left and straight choices, and going from 4 to 3 required straight and right choices (S, R, L, S). We saw that these were the same as for the plus maze, and thus the lapse model gave the same error probabilities. Finally, for the H maze task, the ideal turn probabilities were *P*(left = 1/2), *P*(straight = 0), and *P*(right = 1/2): going from 3 to 4 required two left turns, and going from 4 to 3 required two right turns (R, R, L, L). Applying these probabilities to the lapse model for the plus maze task as above, *P*(choice ≠ 2 | lapse at 3 − 1) = 1/2 and *P*(choice ≠ 3 | lapse at 2 − 1) = 1 and the total alternation error probability became *P*(out error | making out decision and lapse) = 3/4. The reference location error probabilities on lapse trials were *P*(choice ≠ 1 | lapse at 1 − 2) = 1/2 and *P*(choice ≠ 1 | lapse at 1 − 3) = 1, and the total reference location error rate on lapse trials was *P*(in error | making in decision and lapse) = 3/4. This model had the same reference location and alternation error probabilities.

The second strategy we considered was a location preference strategy on lapse trials where the preference for each location depended on the reward rate on the plus maze (Fig. 7, Extended Data Fig. 7-2). The weighted preference of the locations would ideally be [2,1,1,0] based on the alternation rule for Locations 1 through 4 (Location 1 is visited twice as often as 2 and 3 and Location 4 is never rewarded). On a lapse trial based on the location preference, the next location would be drawn proportionally to these weights (with the current location reweighted as 0).

We then computed the probability of the alternation errors on lapse trials: *P*(choice ≠ 2 | lapse at 3 − 1) = 1/2 and *P*(choice ≠ 3 | lapse at 2 − 1) = 1/2. Given that each of the alternation decision types had equal probability, *P*(out error | making out decision and lapse) = 1/2. Similarly, the reference location error probabilities on lapse trials were *P*(choice ≠ 1 | lapse at 1 − 2) = 1/3 and *P*(choice ≠ 1 | lapse at 1 − 3) = 1/3. The total reference location error rate on lapse trials was thus *P*(in error | making in decision and lapse) = 1/3. Under this location strategy, the probability of an alternation error was greater than a reference location error, assuming lapses were equally likely on both decision types.

## Results

To understand how learning a novel task depended on the structure and rules from previous experience, we designed a two-phase spatial learning experiment. We first introduced rats to a differential experience phase, where two groups of rats learned different tasks (Fig. 1*A*). Rats in the “switching” group (*n* = 9) were trained on two mazes each day, the H maze and the 2T maze, counterbalanced for session order (*n* = 9). Rats in the “non-switching” group (*n* = 10) were trained on only the H or 2T maze, twice per day (*n* = 5 for each maze). On both the H and 2T mazes, rats had to visit two of the four arm ends in alternation to receive reward. The “switching” group had an additional requirement to learn alternation rules associated with each maze. To create distinct spatial navigation experiences with similar physical effort, the H and 2T mazes differed in geometry, but the rewarded trajectories had the same path lengths. After Phase 1 training, all animals achieved similar levels of performance in their tasks (Fig. 1*C*).

To understand how these distinct experience histories affected future learning, we then exposed all rats to a new task in Phase 2, which was on plus maze with four locations (1, 2, 3, and 4). To receive reward on every trial, the rats needed to visit three of the four locations in a sequence (1, 2, 1, 3, …; Fig. 1*B*). The plus maze task had an unrewarded fourth arm that was not part of the rule; thus the rat needed to avoid this arm. The alternation rule was analogous to existing spatial alternation tasks (Frank et al., 2000; Kim and Frank, 2009; Jadhav et al., 2012). When the animal was at Location 1, it would be rewarded at Location 2 on the next trial only if it previously visited Location 3, or it would be rewarded at Location 3 on the next trial only if it previously visited Location 2. If the animal was at Locations 2 or 3, it needed to visit Location 1 to be rewarded. These two components reinforced the alternation rule while ensuring reward for partial sequence completion.

Structurally, the mazes in Phases 1 and 2 all contained four arms with reward locations at the ends. The shared features of the mazes were paths, intersections, and reward locations. While these structural features were shared across mazes, the topology and geometry of the paths were distinct. For the Phase 1 mazes, there were two intersections each with three connected arms. For the Phase 2 maze, there was one intersection with four connected arms. On all mazes, the animals needed to decide to turn left or right or go straight at intersections. Although these actions were shared across mazes, the sequence of actions necessary for maximal reward was distinct across mazes. The animals could not directly apply the sequence of turns from Phase 1 tasks to the Phase 2 task.

We asked whether performance on the plus maze, as measured by the reward rate across sessions, depended on the rats’ experience in Phase 1. One prediction was that the two groups would differ in performance, based on findings that diverse experiences could benefit cognitive performance (Petrosini et al., 2009). Alternatively, both groups would show similar performance because neither maze experiences from Phase 1 provided advantages due to the maze layout and reward rule being sufficiently distinct. We did not find statistically significant differences in the reward rate across sessions on the plus maze between experience or task groups (pairwise Wilcoxon rank-sum tests with Benjamini/Hochberg false discovery rate correction: *p* > 0.44 for experience or *p* > 0.83 for task across sessions; Fig. 1*D*).

**Figure 2.**
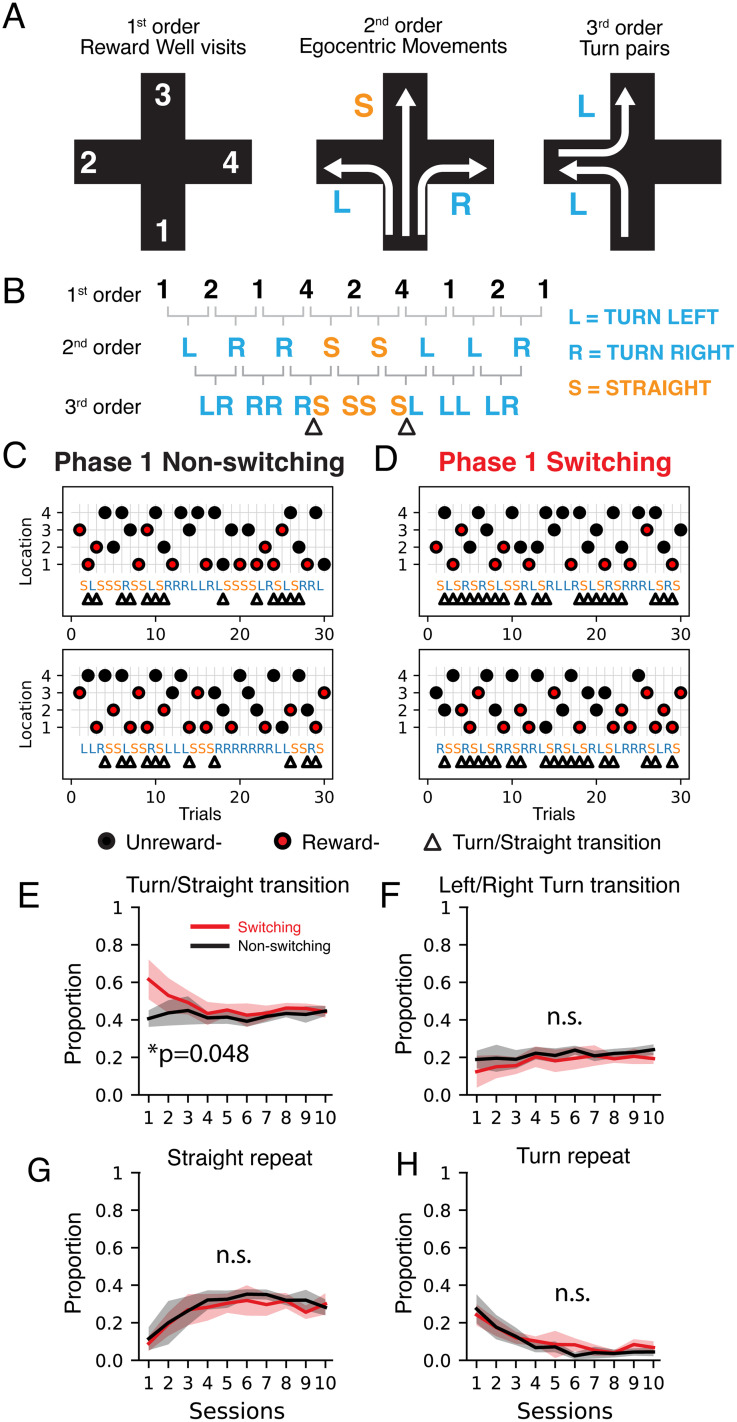
Behavior choice pattern classification for the plus maze. ***A***, Schematic of first-, second-, and third-order description of behavior choices, which were individual location visits, egocentric movements at junctions, and pairs of turns, respectively. ***B***, Example behavior choice sequence and corresponding higher-order descriptions. Triangles indicate a transition between turn and straight actions. ***C***, Example behavior choices for the first 30 trials in the plus maze for two animals in the non-switching group. First-order transitions shown by the circles that indicate the location visited by the rat. Red circles indicate the rewarded visits. Second-order transitions convert the location visit pairs into left turns (L), right turns (R), and straight (S). L and R are marked blue, and S is in orange. Triangles correspond to switch trials or third-order transitions that involve changes between L/R and S. Additional examples in Extended Data Figure 2-1. ***D***, Example behavior choices for the first 30 trials in the plus maze for two animals in the switching group. ***E***, Proportion of turn/straight transition trials (mean and 95% confidence interval of the mean) for each session. These are the frequencies of the trial pairs marked by triangles in ***C*** and ***D***. Wilcoxon rank-sum test with Benjamin/Hochberg false discovery rate correction, *p* = 0.048 for the first session only. ***F***, Proportion of transitions between left and right turns (mean and 95% confidence interval of the mean) for each session. Wilcoxon rank-sum test not significant. ***G***, Proportion of repeated straight choices (mean and 95% confidence interval of the mean) for each session. Wilcoxon rank-sum test not significant. ***H***, Proportion of repeated turn choices (mean and 95% confidence interval of the mean) for each session. Wilcoxon rank-sum test not significant.

We reasoned that there may have been differences in the rats’ behavior that were not captured by only quantifying the reward rate. Because the tasks required the animals to sequentially visit locations, we hypothesized that the choice patterns (Fig. 2*A*,*B*) could reflect distinct strategies used to solve the problem and could provide further insight into the learning process. We quantified patterns of action choices because all mazes in the experiment had intersections allowing for turns and/or going straight. Conversely, spatial features, such as relative distance between the reward locations, were not directly comparable between the Phase 1 and 2 mazes. We defined first-order behavior as the sequence of locations visits. Second-order behavior captured the egocentric action required to travel between locations: left turn (L), straight (S), or right turn (R). We further classified these third-order action pairs into transitions between actions (turn/straight or between turns) or repeating actions.

10.1523/ENEURO.0266-24.2024.f2-1 Figure 2-1 Behavior choices for the first 30 trials of the Plus maze for all animals, shown in the same format as Fig. 2 C-D. 1^st^ order transitions shown by the circles that indicate the maze location visited by the rat. Red circles indicate the rewarded visits. 2^nd^ order transitions convert the location visit pairs into left turns (L), right turns (R) and straight (S). L and R are marked blue, and S is in orange. Triangles correspond to switch trials, or 3^rd^ order transitions that involve changes between L/R and S. Download Figure 2-1, TIF file.

We found that animals in the switching group made more turn/straight transitions compared with the non-switching group (Fig. 2*C*–*E*, Extended Data Fig. 2-1, switch trials indicated with triangles). This difference was significant only for the first session (Fig. 2*E*). There were no differences between groups in the frequencies of transitions between turns or repeated actions (Fig. 2*F*–*H*).

**Figure 3.**
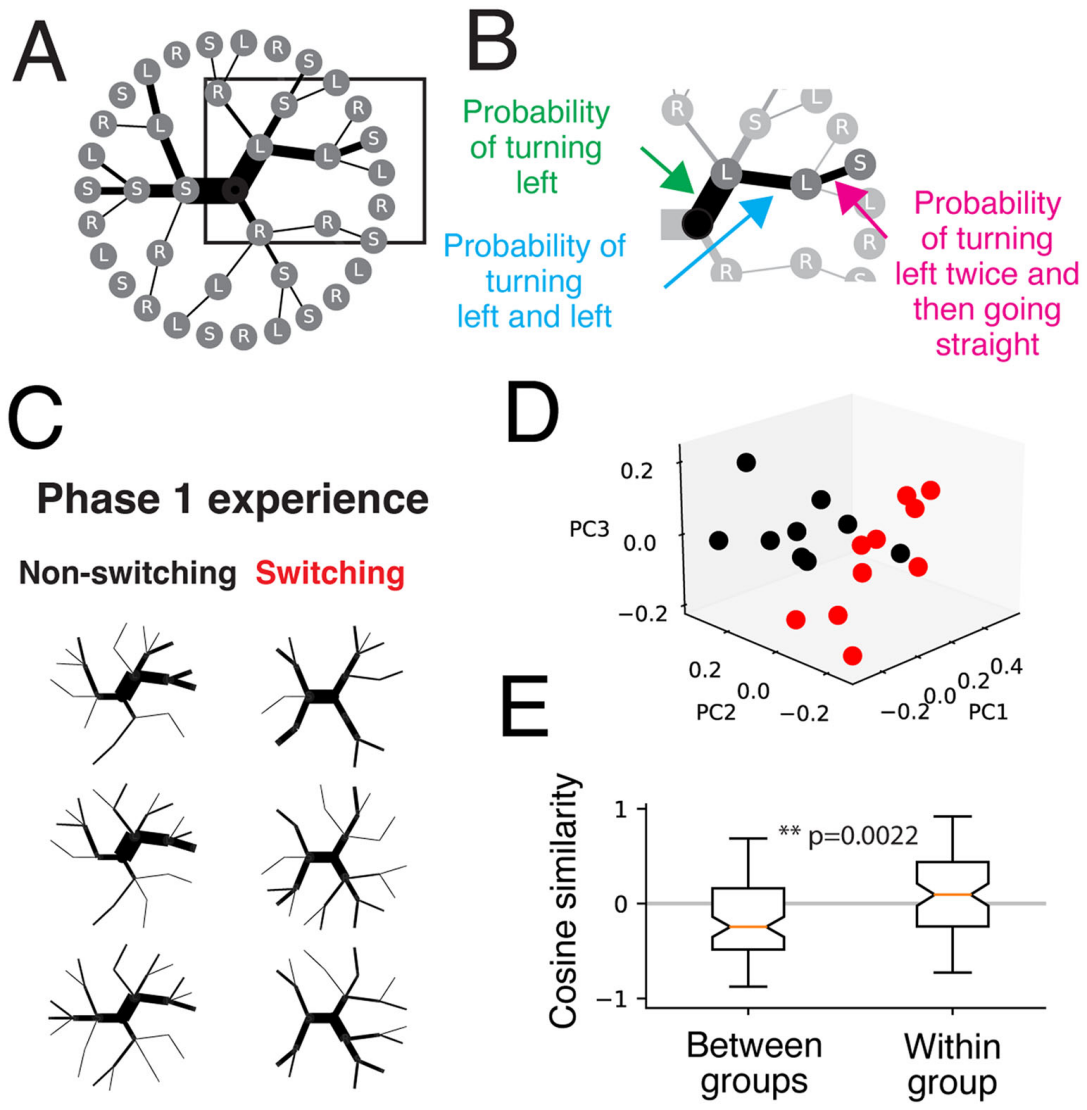
Visualizing sequential behavior choice probabilities. ***A***, Example dendrogram of conditional probabilities for three-trial choice sequences. Edges represent the conditional probability, and nodes represent the choices. ***B***, Example showing the probabilities of sequences with one, two, or three trials. ***C***, Choice probability dendrograms for the first 30 trials of the plus, H and 2T mazes. Three example animals from the non-switching (left column) and switching (right column) are shown. ***D***, Scatter plot of the first three principal components of the choice probabilities between non-switching (black) and switching (red) groups for the first 30 trials on each maze. ***E***, Cosine similarity across all principal components of choice probabilities between groups and within group for the first 30 trials. Wilcoxon rank-sum test *p* = 0.0022.

To gain further insight on each animal's choice sequences, we examined the choices patterns in intervals of three trials. Given that sequential choice probability arrays are difficult to visually inspect, we found dendrograms provided an intuitive visualization (Fig. 3*A*), where each node represented a choice and the connected nodes were subsequent choices (Fig. 3*B*). The thickness of the edge connecting two nodes indicated the probability of that choice, where thicker lines indicated higher probability (see Materials and Methods).

The sequential choice probability dendrograms revealed distinct patterns between experience groups during the first 30 trials, corresponding to early learning in the plus maze (Fig. 3*C*). To visualize and quantify differences between patterns across the two experience groups, we applied PCA to the sequential choice probability array (Fig. 3*D*). The scatter plots of the first three principal components showed a separation between the non-switching and switching groups. We confirmed this separation by calculating the cosine similarity between the principal components of rats. We found that the distance between the non-switching experience rats and switching experience rats was greater than the distance between rats within each experience group, non-switching or switching (Fig. 3*E*). This was consistent with the idea that non-switching and switching experience groups differed in choice sequence patterns.

**Figure 4.**
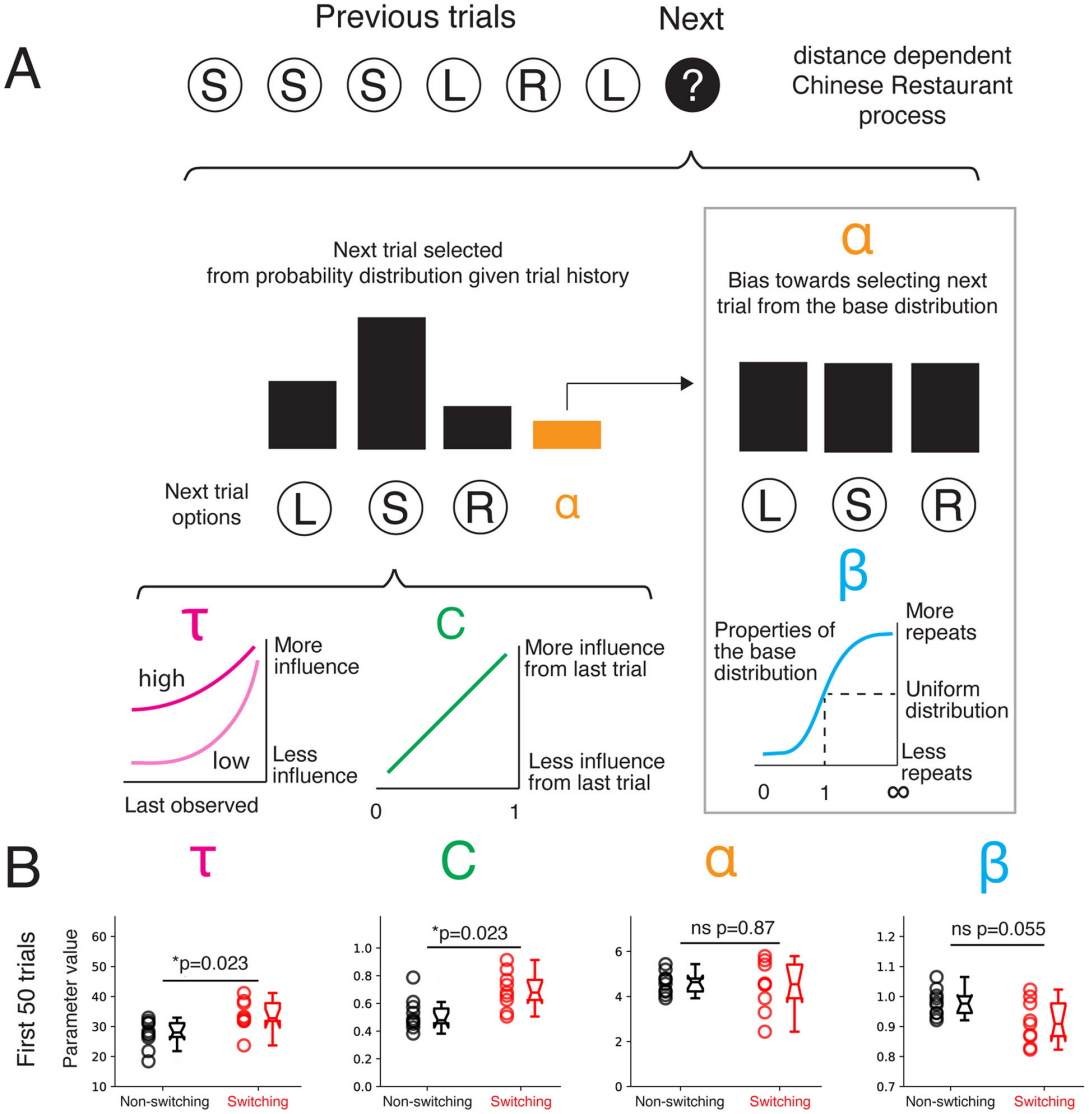
Models of non-switching and switching groups show distinct parameters that control how past trials influence future trials. ***A***, Schematic of a modified ddCRP model. *τ* modulates the time-dependent influence of all previous trials on the next trial, effectively controlling the decay rate over all previous trials. Larger values of *τ* correspond to a more persistent influence of previous choices on the next choice. *C* modulates the dependence of the next trial on the immediate previous trial. Higher values of *C* correspond to a greater influence of the previous trial on the next trial. *ɑ* determines likelihood the next trial is drawn from a base distribution instead of trial history. *β* determines the likelihood the base distribution is governed by a uniform distribution (*β* = 0) or a distribution that is biased to repeat (*β* > 0) or avoid previous choices (*β* < 0). Model validation in Extended Data Figure 4-1. ***B***, Scatter- and boxplots of model parameters from model fit to each animal's first 50 trials on the plus maze. Wilcoxon rank-sum test with Benjamini/Hochberg false discovery rate correction for *τ* (*p* = 0.023), *C* (*p* = 0.023), *ɑ* (*p* = 0.87), and *β* (*p* = 0.055).

We next asked what differences in the underlying processes could give rise to these distinct behavior patterns. We aimed to characterize the structure of the rats’ choice sequences during the early learning period in the plus maze. We therefore fitted statistical models with a small set of interpretable parameters that related choice history with future choices using the observed choice sequences for each rat. We then determined whether models from each experience group had different estimated parameter values. Between-group differences in model parameters could reveal differences in the statistical processes that generated the sequences. We chose the ddCRP (Blei and Frazier, 2011), which assumed each choice in a sequence was sampled from a distribution of possible choices that was dependent on past choices on two different timescales (Fig. 4*A*). The model contained a time constant (*τ*) that determined how influential all past choices were on the next choice, as modeled by an exponential decay, which allowed for changes in behavior during early learning. Given that we found differences in the likelihood of switching between different choices, we added a parameter that determined the relative influence of the previous choice on the next choice (*C*). The model also included a parameter that determined how closely the probability of choices was concentrated toward a “base” distribution (*ɑ*). The base distribution was parameterized to account for simple behavioral strategies based on varying likelihood of repetition (*β*) without taking more specific trial history into account. Our goal was to compare features of behavioral patterns observed within each experience group during the early learning period by fitting the model to each animal's behavior during the first 50 trials and comparing the fitted parameters across groups. We performed simulations to show differences in parameters could be recovered from the models (Extended Data Fig. 4-1).

10.1523/ENEURO.0266-24.2024.f4-1 Figure 4-1 Simulations confirm that the model parameters can be recovered from sequences of actions. We simulated from the distance-dependent Chinese restaurant process using two different sets of parameters (simulations 1 and 2, dashed lines indicate the true parameters). For each set of parameters, we generated 50 independent simulations. The parameters were then fit with an increasing number of trials using the posterior median as the estimate. The points give the mean estimates, and the error bars show a 90% interval over simulations. The estimated parameters remained close to the prior distribution with few trials and tended towards the true parameters with increasing amounts of data. We found that the context dependency parameter (C) required the fewest number of trials to separate across these two simulations. Given low values of the chosen base distribution bias (ɑ), which meant the base distribution was unlikely to be chosen in the generated sequences compared with the history-dependent distributions, we did not expect the repetition bias parameter (β) to be effectively recovered. Download Figure 4-1, TIF file.

The statistical model fits showed between-group differences in how previous choices were related to future choices. The non-switching and switching groups differed in the time constant (*τ*), where the switching group had a significantly longer time constant, indicating that past choices were more predictive of future choices in the switching group (Fig. 4*B*). The two groups also differed in how the immediate past affected the upcoming choice (*C*): the previous choice had a stronger influence for the switching group compared with the non-switching group. This finding supports our previous observation that the groups differed in how often they switched between turning and not turning (Fig. 2*E*), and it points to differences in how each group used working memory during exploration to switch to a different action than before. The bias toward selecting the next trial from the base distribution (*ɑ*) was not significantly different between the two groups. The base distribution trended toward having less repetition (*β*) in the switching compared with the non-switching group.

**Figure 5.**
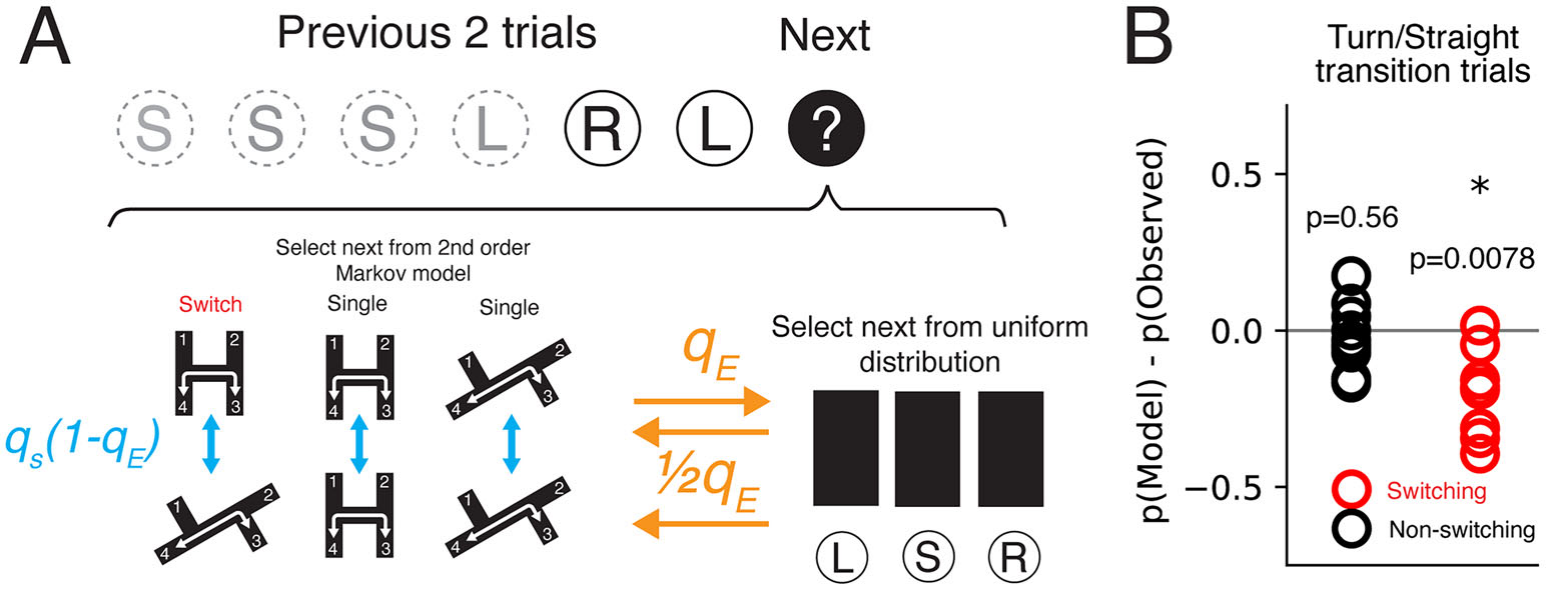
Learned action patterns could not explain choice sequences in new task. ***A***, Simulating choice sequences in Phase 2 based on learned action sequences from Phase 1. The previous two trials determined the distribution from which the next choice was selected. The second-order Markov models were trained on the observed action sequences from each maze in Phase 1. At each step, the model determined whether to select the next choice from the Phase 1 action distributions or from a uniform distribution (*q*_E_). If the Phase 1 distributions were chosen, the model selected the next choice from one of the two maze distributions (*q*_s_). ***B***, Difference in probability of total turn/straight transition trials between simulation and observed behavior.

We next sought to understand which aspects of the rats’ Phase 1 experience could explain their exploratory choice sequences in Phase 2. One possibility is that the animals were applying Phase 1 action sequences to Phase 2. To test this hypothesis, we constructed a model to simulate location visit sequences on the plus maze based on learned action sequences from mazes in Phase 1. The Phase 1 turn sequence was modeled by a second-order Markov chain built on each rat's choice sequences from the last two sessions of Phase 1. We included a parameter that controlled the likelihood that the next choice was selected from learned action sequences or from a uniformly random choice (Fig. 5*A*), which added flexibility to choose between known rules or to discard known rules in favor of random exploration. We found this model could not capture observed choice sequence properties in Phase 2 across a range of parameters that controlled the selection of the next location visit (Fig. 5*B*). Models produced a lower likelihood of turn/straight transition trials switching group compared with observed data (Fig. 5*B*). Thus, the observed exploratory behavior could not be explained by applying learned action sequences from Phase 1 or switching between learned sequences, even with allowance for more random exploratory choices.

**Figure 6.**
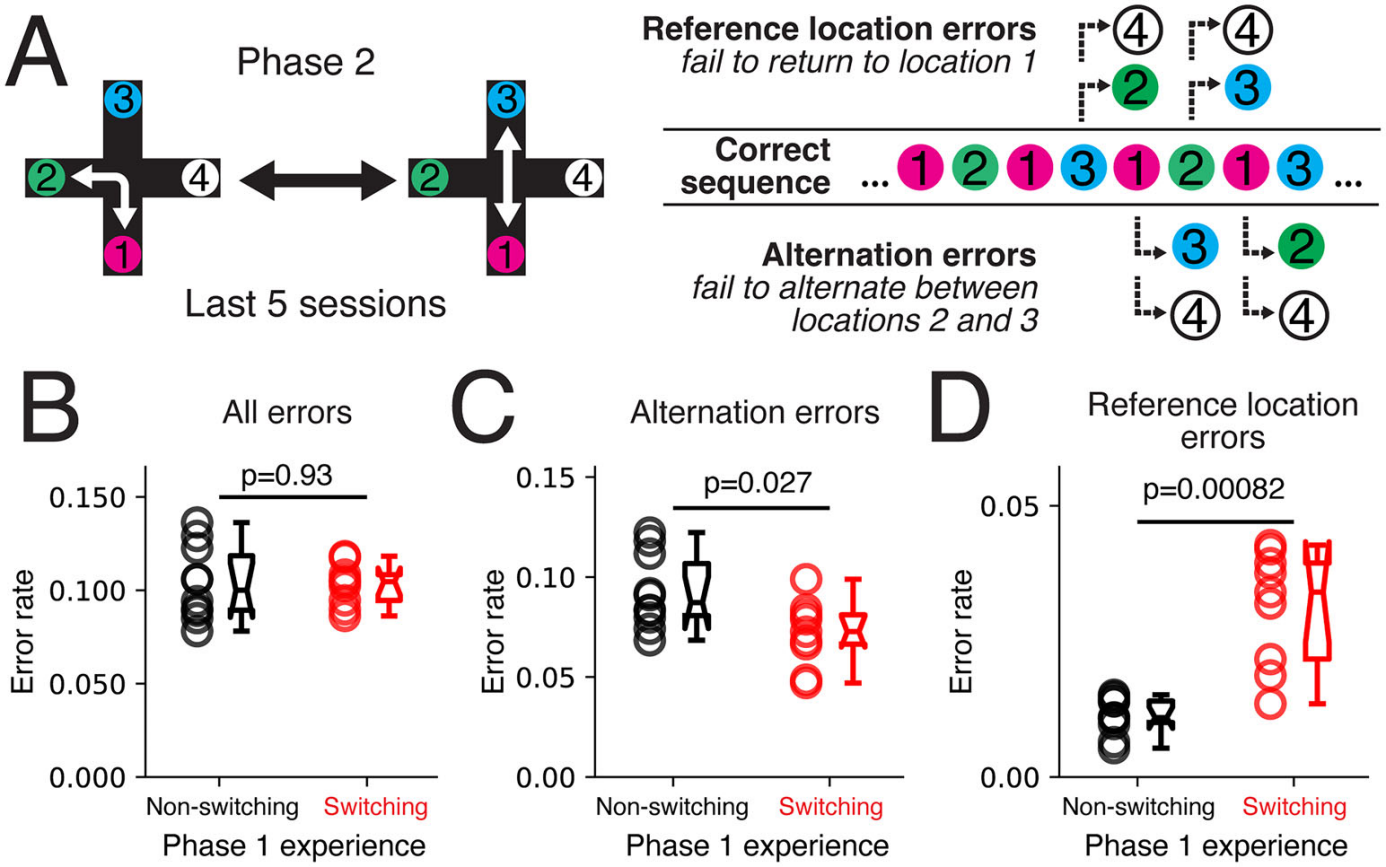
Non-switching and switching groups showed distinct errors during stable performance. ***A***, Schematic of error types. Reference location errors were out-of-sequence choices after visiting Locations 2 and 3. Alternation errors were out-of-sequence choices after visiting Location 1. Errors were defined as the first out-of-sequence choice after correctly completing a sequence of four visits. ***B***, Similar rates of all errors, both reference and alternation, for the last five behavior sessions. Wilcoxon rank-sum test *p* = 0.93. ***C***, Non-switching group animals showed higher rates of alternation errors compared with switching group animals for the last five behavior sessions. Wilcoxon rank-sum test *p* = 0.027. ***D***, Switching group animals showed higher rates of reference location errors compared with non-switching animals for the last five behavior sessions. Wilcoxon rank-sum test *p* = 0.00082.

So far, we saw distinct exploratory patterns between the two experience groups; we next tested whether behavioral differences persisted even when performance plateaued in later sessions. We reasoned that the plus maze task could be solved with different strategies. Each strategy may have had its own strengths and weaknesses, producing distinct types of errors. Errors occurred in our task and in related continuous spatial alternation tasks, even once the animal's performance plateaued (Fig. 1*D*; Kim and Frank, 2009; Jadhav et al., 2012; Singer et al., 2013; Kastner et al., 2020, 2022). In these continuous alternation tasks, performing a correct sequence of choices (Fig. 6*A*) has been hypothesized to rely on two distinct memory mechanisms (Olton et al., 1979; Kim and Frank, 2009; Jadhav et al., 2012). When the rat departed from Location 1, it needed to visit Locations 2 or 3, but not the previously visited one (Fig. 6*A*). This decision to alternate was thought to rely on integrating working memory of the immediate past choice with the current location. We refer to errors on these trials as “alternation errors.” In contrast, when the animal was at Locations 2, 3, or 4, it always needed to return to Location 1 (Fig. 6*A*). This decision to return to a fixed location was thought to rely on reference location memory, where the next choice should be Location 1 on the maze irrespective of the current location. We refer to errors on these trials as “reference location errors.” By analyzing the error rate at each of these steps, we could understand the reliance of any strategy on these distinct memory types.

We found that the two experience groups differed in alternation and reference location error rates, despite having similar overall error rates (Fig. 6*B*–*D*). For the last five sessions in Phase 2, when the performance was ∼80% (Fig. 1*D*), we quantified error rates after the rat completed a sequence of four correct location visits (Fig. 6*A*). This type of error reflected a sudden lapse after a bout of correct performance. Intriguingly, we found opposite rates of alternation and reference location error rates between the two experience groups. The switching experience group made fewer alternation errors than the non-switching group, indicating that the switching experience rats were better at correctly alternating between Locations 2 and 3 (Fig. 6*C*). The improved alternation performance was consistent with the switching group's exploratory behavior during early learning (Fig. 2*E*), where they switched between turning and going straight more frequently than the non-switching group. The non-switching group made fewer reference location errors, indicating they were better at consistently returning to Location 1 (Fig. 6*D*). These results suggested the switching group's strategy relied more on working memory, whereas the non-switching group’ strategy relied more on reference location memory.

**Figure 7.**
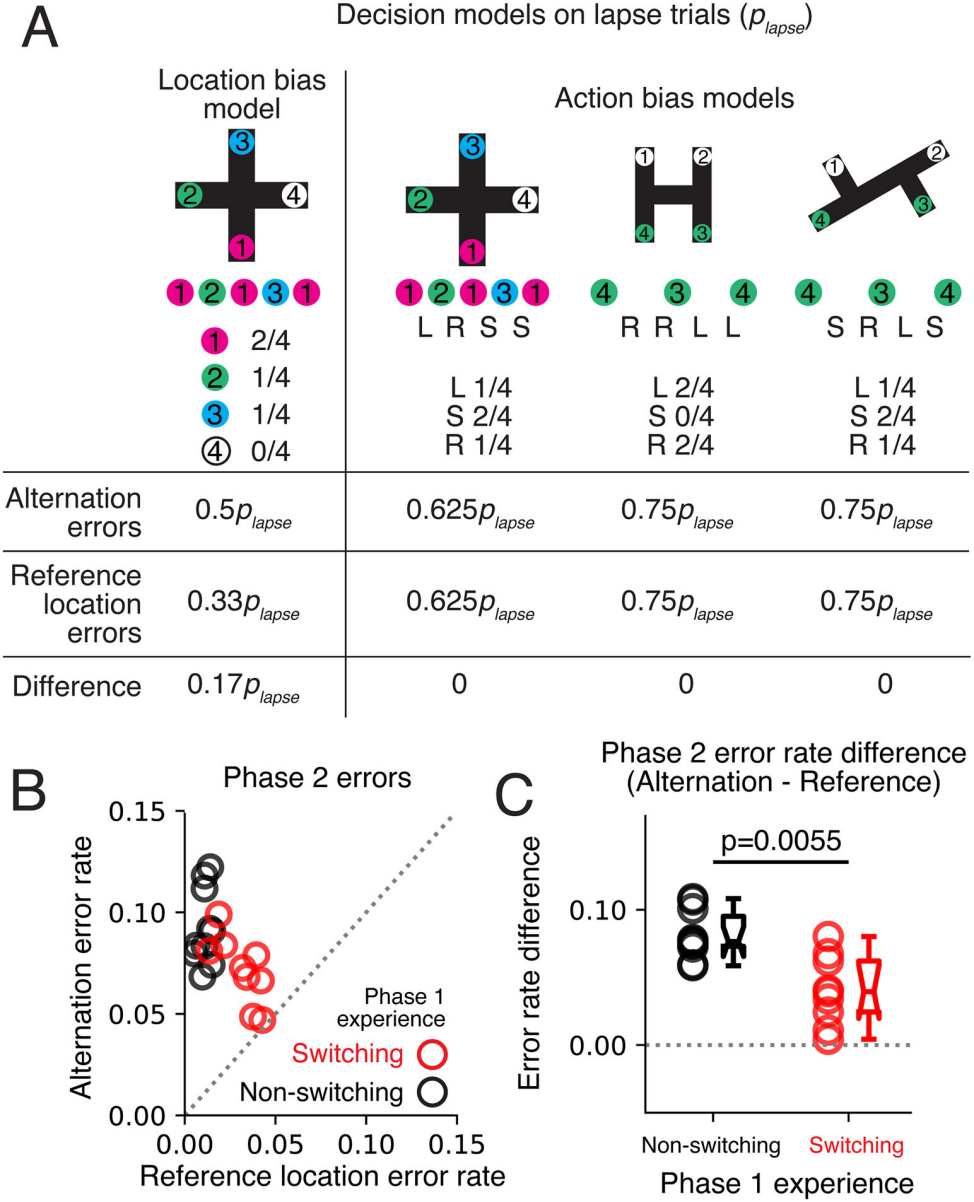
Non-switching group errors consistent with a location-bias strategy. ***A***, Expected error rates based on lapse trials following a location or action bias. The location bias assumes rats default to visiting the location most frequently rewarded in the task. This model predicted higher alternation compared with reference location error rates. The turn bias model assumed rats default to following a sequence of turns based on learned turn sequences from all tasks. These models predicted similar rates of alternation and reference location errors. Details of calculations in Extended Data Figures 7-1 and 7-2. ***B***, Scatterplot of alternation versus reference location errors. ***C***, Difference between alternation and reference location errors. The non-switching group showed a greater difference between alternation and reference location errors. Wilcoxon rank-sum test *p* = 0.0055.

We postulated that the difference in alternation and reference location error rates across the two groups could be explained by different strategies that lead to different outcomes on lapse trials (Fig. 7, Extended Data Figs. 7-1, 7-2; see Materials and Methods: origin of errors based on error-type probabilities). We assumed that, on lapse trials, the action would be driven by a simple underlying bias. We constructed lapse strategies based on the tasks and computed the error rate. First, a location-bias strategy selected the next choice based on the most frequently visited location for the plus maze (Fig. 7*A*). Because a larger fraction of rewards was associated with Location 1, this hypothesis predicted a higher alternation versus reference location error rate (Fig. 7*A*, Extended Data Fig. 7-2). Second, in an action-biased strategy, the next action was selected from the average action probabilities (left, straight, or right) on any maze the rats have learned (Fig. 7*A*). This hypothesis predicted similar reference location and alternation error rates, irrespective of the maze action sequence. The overall alternation error rate was higher than the reference location error rate (Fig. 7*B*), which was consistent with previous findings (Kim and Frank, 2009; Jadhav et al., 2012). Furthermore, we found the non-switching group had a larger difference between the alternation and reference location error rates (Fig. 7*C*). This was consistent with the non-switching group having a stronger place bias compared with the switching group.

10.1523/ENEURO.0266-24.2024.f7-1 Figure 7-1 Error likelihood predicted by a turn-bias strategy. Download Figure 7-1, TIF file.

10.1523/ENEURO.0266-24.2024.f7-2 Figure 7-2 Error likelihood according to a location-bias strategy. Download Figure 7-2, TIF file.

The opposing error rates between the non-switching and switching groups linked each group's Phase 2 strategy back to their experience in Phase 1. Rats in the non-switching group learned to consistently alternate between two fixed locations in Phase 1. This strategy of revisiting fixed locations benefited the reference location component of Phase 2. However, the reliance on reference location memory impaired the alternation component of the task in Phase 2, where avoiding the previously visited location was needed. In contrast, the switching group had to learn two distinct maze rules in Phase 1. The flexibility to switch between contexts and rules was advantageous for the action switching that was necessary for the alternation component of Phase 2. However, this switching flexibility impaired the reference location component of plus maze in Phase 2, where returning to a fixed location was necessary.

These findings suggested the new task was learned by modifying existing strategies from prior experience. Although the maze structures between the two experiment phases shared some similarities, how this knowledge was applied to performing the task on each maze differed. The strategies for solving the tasks were more likely to be transferrable. Our results showed the strategies from Phase 1 were influencing the choices made in Phase 2 and the impact of prior experience remained persistent throughout the learning process as well as performing the task after learning.

## Discussion

There are multiple mechanisms through which prior experience can influence future behavior. Transfer and generalization involve integrating the memories of multiple experiences to form a broader representation of the concepts shared between those experiences. When learned rules could be generalized to new problems, the new problem could be solved faster (Webb, 1917). However, instead of performance enhancements, we observed differences in navigation choices during exploration and the types of errors made during stable performance. Our findings revealed that even when memory of space and rule may not directly apply in a new task, other aspects of the animals’ experience histories could influence how they learn and perform the new task. This indicated that transfer can occur through problem-solving strategies rather than specific learned rules. These strategies could span multiple timescales. In Phase 1, the rule remained the same across trials, but the switch in rules occurred across sessions for the switching group.

The rats’ experiences in Phase 1 reinforced different strategies for spatial navigation, determining how they first approached exploring the new plus maze at the start of Phase 2. Throughout training on the plus maze, their performance converged to follow the new rule, ending with similar overall performance rates. However, hidden within this similar performance were between-group differences in the types of errors, indicating that the rats relied on different strategies influenced by their experience in Phase 1. For a strategy that relied on reference location memory, the reward locations for both phases could be memorized as a sequence of locations, two for Phase 1 and four in Phase 2. For Phase 2, greater reliance on this reference memory could create a stronger preference for Location 1 as the home base destination, creating an advantage for returning to Location 1. This was what we saw in the non-switching group, which in Phase 1 were only required to repeatedly visit two fixed locations. For a strategy that relied on flexibly switching between different locations, the tasks in both phases could instead be learned as point-to-point alternations. Phase 1 was one single point-to point-alternation for the non-switching group or two single point-to-point alternations for the switching group. Phase 2 was switching between two point-to-point alternations. To switch between alternations on the plus maze in Phase 2, the rat would need to apply working memory to base their next action on the previous action. This indicated that the switching group may have applied a strategy with greater reliance on the use of working memory to alternate between behaviors than the non-switching group, leading to fewer alternation errors at the cost of more reference location errors.

An important direction for future research is to understand how experience shapes neural processes for learning. Our results pointed to experience-dependent reliance on spatial working memory or reference location memory, which may engage distinct brain networks. Even though distinct brain regions, such as cortical and hippocampal regions, are involved in spatial learning, prior experience may alter the relative contribution of these regions when making decisions in the future. This suggests that prior experience may change the underlying computations used for discovering and applying new rules during learning. For spatial navigation tasks, both reference and spatial working memory involve the hippocampus (Olton et al., 1979). Hippocampal lesions slow the acquisition of reference location and alternation rules (Kim and Frank, 2009). Interruptions to hippocampal sharp wave ripples impair the spatial working memory component while leaving the reference memory component of the task intact (Jadhav et al., 2012). Functional disruption of the hippocampus and prefrontal cortex also impairs spatial working memory (Maharjan et al., 2018). Recent findings show that neural representations of decision-making tasks are dependent on experience (Latimer and Freedman, 2023). Animals with different training histories have distinct cortical task dynamics even when performing the same task with similar behavior outcomes. Frontal cortical and hippocampal networks are implicated in representing task rules and modulating generalization (Winocur and Moscovitch, 1990; Freedman, 2001; Wallis et al., 2001; Rich and Shapiro, 2009; Tse et al., 2011; Wang et al., 2012; Xu and Südhof, 2013; Morrissey et al., 2017; Yu et al., 2018; Kaefer et al., 2020; Samborska et al., 2022). These networks could modulate strategic decisions when solving new problems. When previously learned rules cannot be successfully applied, these networks could discard the application of existing rules in favor of more flexible choice policies (Karlsson et al., 2012; Tervo et al., 2014). The animal's experience may influence which types of polices are favored. We speculate that each experience has distinct effects on nodes in hippocampal-cortical networks, resulting in distinct decision-making strategies that are experience-dependent. At any moment, the state of the network is a product of the individual's unique experience history. We hypothesize that the experience-dependent configuration of the network provides priors and neurocomputational building blocks for generating new behaviors unique to each individual.

In our experiment, experience with switching between tasks benefited components of learning that required switching, while experience with only reference location memory tasks benefited components of learning that required memorizing fixed locations. Our results suggest that unique experience histories create unique starting points for how individuals approach new situations. The impact of prior experience extends from the strategies used to discover new rules and how the new task is performed after the task is learned. This demonstrates that the same task can be learned and performed in multiple ways, and the impact of prior experience can persist into the future.

## Synthesis

Reviewing Editor: Mark Laubach, American University Decisions are customarily a result of the Reviewing Editor and the peer reviewers coming together and discussing their recommendations until a consensus is reached. When revisions are invited, a fact-based synthesis statement explaining their decision and outlining what is needed to prepare a revision will be listed below. The following reviewer(s) agreed to reveal their identity: Douglas Nitz, Alejandra Alonso. Note: If this manuscript was transferred from JNeurosci and a decision was made to accept the manuscript without peer review, a brief statement to this effect will instead be what is listed below.

Thank you for submitting your work to eNeuro!

You manuscript was reviewed by two experts. Their reviews are given below in full. Please revise to address all points that were raised.

Please also consider submitting a visual abstract. This helps with promotion of your study by the journal.

Reviewer 1

Awh et al. examine differences in a "phase 2" plus maze navigational task in which animals must repeatedly return to a single arm between trajectories to two other arms in alternation. Two groups are defined in "phase 1" according to whether they were exposed to moving between two arms of an H maze or a more irregularly-shaped maze ("non-switchers") versus or moving between two arms of the H maze and the more irregularly shaped maze ("switchers"). During "phase 2", switchers make more "reference memory" errors (failure to return to the single arm between visits to other arms) while non-switchers make more "working memory" errors (failure to alternate when moving away from the single arm). The task design is set up to test whether the two groups will differ despite the irrelevance of the phase 1 tasks to learning and performance on the phase 2 task.

The analytical approaches are thorough and appropriate and the statistical differences between groups seem robust. The approach does seem to over-emphasize analysis using rather complex models only to arrive at results and conclusions that could be reached with simpler, more direct quantifications of choice behavior.

The description of the phase 2 task could be improved. It is not clear what the rule is exactly. If the animal alternates between arm 2 and 3 (with returns to 1 in-between), is the case that arm 4 never needs to be visited?

All 3 mazes have the same basic structure (4 arms with a connecting intersection that is point-like (phase 2) or is a short path segment (phase 1). Yet the authors claim that there is nothing about the phase 1 experience that can inform the phase 2 experience. Further, the animals alternate, in some form, in all 3 mazes. The "reliance" on working memory posited for the switchers might simply reflect having had a second experience in an alternation task (on a different maze). It would help for the authors to more clearly speak to the similarities between the mazes and the task and the similarities between shuttling tasks and alternation tasks. This is not to say that the tasks aren't different, but to acknowledge that they may be more different to the human experimenter using black-box terms for types of memory than to the rat itself.

While the work seems to be well done, the conclusions one can reach are somewhat limited. The differential experience in phase 1 predicts some differences in phase 2 despite, as the authors claim, the idea that the phase 1 tasks are irrelevant to phase 2 learning and performance. Even if one takes the claim of irrelevance as true (I, for one, do not), there is still the question of why the combined experience on two mazes in phase 1 yields phase 2 differences. As it stands, the main claim is simply that they do. The manuscript could be improved in this sense.

Reviewer 2

In Figure 2C, it is unclear why all animals start with a reward in locations 2 or 3, except for the last animal, which also starts at location 3 but is not rewarded. Unlike the other examples, the last animal did not proceed to location 1. However, it is still unclear why it was not initially rewarded like the other animals.

Further, in the same figure, for the first animal of the non-switching group, animals are rewarded when they visit 3-1-2-1-3-1 with some unrewarded choices in between. However, the subsequent three visits to location 1 are rewarded. Does this imply that location 1 continues to be rewarded until the next correct choice is made? The example graphs need verification, or the description of reward delivery requires clarification.

Additionally, Figure 4 would benefit from improved legends within the figure, to make it easier to understand tau, c, alfa and beta, in particular tau.

Minor issues

In Figure 2, the labels for letters F and H are not fully printed.

Line 433 refers to Figure 8A, which appears to be a mistake. It should refer to Figure 6A.

Line 464 refers to Figure 9A, which appears to be a mistake. It should refer to Figure 7A.

## Author Response

Reviewer 1 Awh et al. examine differences in a "phase 2" plus maze navigational task in which animals must repeatedly return to a single arm between trajectories to two other arms in alternation. Two groups are defined in "phase 1" according to whether they were exposed to moving between two arms of an H maze or a more irregularly-shaped maze ("non-switchers") versus or moving between two arms of the H maze and the more irregularly shaped maze ("switchers"). During "phase 2", switchers make more "reference memory" errors (failure to return to the single arm between visits to other arms) while non-switchers make more "working memory" errors (failure to alternate when moving away from the single arm). The task design is set up to test whether the two groups will differ despite the irrelevance of the phase 1 tasks to learning and performance on the phase 2 task. The analytical approaches are thorough and appropriate and the statistical differences between groups seem robust. The approach does seem to over-emphasize analysis using rather complex models only to arrive at results and conclusions that could be reached with simpler, more direct quantifications of choice behavior. The description of the phase 2 task could be improved. It is not clear what the rule is exactly. If the animal alternates between arm 2 and 3 (with returns to 1 in-between), is the case that arm 4 never needs to be visited? We thank reviewer 1 (R1) for identifying areas for clarification. R1 is correct, arm 4 never needs to be visited. To be rewarded on every trial the rat needs to repeatedly visit locations in the sequence 1, 2, 1, 3. Thus, the correct sequence involves only locations 1, 2, and 3, location 4 is not part of the sequence. We have updated the description of the task in the Methods and the Results. All 3 mazes have the same basic structure (4 arms with a connecting intersection that is point like (phase 2) or is a short path segment (phase 1). Yet the authors claim that there is nothing about the phase 1 experience that can inform the phase 2 experience. Further, the animals alternate, in some form, in all 3 mazes. The "reliance" on working memory posited for the switchers might simply reflect having had a second experience in an alternation task (on a different maze). It would help for the authors to more clearly speak to the similarities between the mazes and the task and the similarities between shuttling tasks and alternation tasks. This is another area we have now clarified based on the suggestion from R1. We have more clearly described the similarities as well as differences in the structure and rules between the mazes and the tasks (lines 352-359). We apologize if we created the impression that Phase 1 experience is irrelevant, that was not our goal. Our goal was to examine differences in experience histories on learning by creating distinct prior task experiences. We have edited the text to remove statements assuming the animals could not use information from Phase 1 to solve the Phase 2 task. This includes the title, abstract and text to emphasize these points. Structurally, the tasks in Phases 1 and 2 are both elevated mazes containing four arms with reward wells at the ends. The shared features of the mazes are paths, intersections and reward locations. For phase 1 mazes, there are two intersections each with three connected arms. For phase 2, there is one intersection with four connected arms. Thus, how the paths are connected and the locations of reward relative to the intersections are distinct. Task wise, the rules can be viewed as a sequence of location visits or a sequence of choices at intersections. For the tasks in Phase 1, the rat needs to alternate between two of the four terminal locations on the maze. From an action sequence perspective, this involves a distinct set of choices at each of the two intersections on each the maze. For the task in Phase 2, the rat needs to perform two sets of alternations across three of the four arms. All mazes require the animal to make decisions at the intersections (left, right or straight) but the action sequences involved are distinct between the mazes. R1 is correct about the tasks having structural similarities that may allow the rat to use information from Phase 1 to learn the task in Phase 2. These are general aspects of the environment, such as the existence of paths, intersections and reward locations. The mapping between these aspects of experience and task rule is distinct between tasks in the two phases of the experiment. Thus, the experimenter-imposed rules as well as the animal's actions to receive reward are distinct between the two phases. We also want to contrast our task design with experiments that investigate generalization of task rules. One type of generalization experiment will have the identical task rule applied in a new context. For example, the same maze or task in a different environment. Similarly, another approach is to rearrange reward locations in the same environment by rotating the maze or making available a different set of locations for reward, thus the learned rule remains the same. Our experiment design is different to those approaches. We alter the topology of the environment, and the rules for the task in both phases are distinct. This is not to say that the tasks aren't different, but to acknowledge that they may be more different to the human experimenter using black-box terms for types of memory than to the rat itself. We agree with R1 that black box terms like "working memory" or "reference memory" may not adequately capture the processes that give rise to these differences. We have removed these assumptions in earlier parts of the Results section and focused on similarities and differences of the task structure and rule. We instead discuss these as possible explainer later in the results. These discussions are in the context of existing concepts and findings from maze-based experiments as cited in the text (Olton et al., 1979; Kim and Frank, 2009; Jadhav et al., 2012). These previous findings support distinct memory processes being involved in different components of maze alternation tasks, which have informed our maze designs. These frameworks provide one way to interpret our results, which we propose as the most parsimonious. We ruled out explanations for observed navigation differences between groups based on repeating a sequence of learned actions (Fig. 5). We also provided statistical tests to explain the observed error patterns under different navigation strategies (Fig. 7). While the work seems to be well done, the conclusions one can reach are somewhat limited. The differential experience in phase 1 predicts some differences in phase 2 despite, as the authors claim, the idea that the phase 1 tasks are irrelevant to phase 2 learning and performance. Even if one takes the claim of irrelevance as true (I, for one, do not), there is still the question of why the combined experience on two mazes in phase 1 yields phase 2 differences. As it stands, the main claim is simply that they do. The manuscript could be improved in this sense. We appreciate R1's suggestion and have clarified the discussion on the conclusions we can draw from these results. We have now clarified which aspects of the tasks are similar (see previous response). In addition, we are now emphasizing an important finding in our results: the effect of prior experience persists as the animal learns a new task and surprisingly even after performance has stabilized. This indicates prior experience history determines how the same task is learned and performed. This provides evidence that new learning can build on rather than override existing knowledge. Our results show that there are differences in the rats' choices both in the beginning of Phase 2 training and in the end of Phase 2, during stable performance. The difference at the start of learning in Phase 2 is likely due to distinct Phase 1 priors that are applied to a novel environment with partially shared features. This could come from the structural layout of the mazes as R1 has pointed out. However, when the animals have learned the task in Phase 2, as indicated by a plateau in performance, the animals still showed signs of experience-dependent differences in the errors made. These errors can be related to their prior experience in Phase 1. Further, we provide a hypothesis for why the "combined experience on two mazes in phase 1 yields phase 2 differences". This is in the last sections of the results. Rats in the non- switching group learned to consistently alternate between two locations in Phase 1. This strategy of revisiting locations benefited "return to location 1" component of the Phase 2 rule. However, the reliance on returning to a fixed location impaired the alternation component of the task in Phase 2, where avoiding the previously visited location was needed. In contrast, the switching group had to learn two distinct maze layouts in Phase 1, requiring them to switch between contexts. We propose that the flexibility to switch between contexts is advantageous for the alternation component of Phase 2. However, this switching flexibility came at a cost the return to location 1 component of Plus maze in Phase 2. We agree with R1's previous comment from above "The "reliance" on working memory posited for the switchers might simply reflect having had a second experience in an alternation task (on a different maze)." We propose the context switching is the benefit gained by the switching group in Phase 1. This is interesting since the timescale for this switching is over sessions in Phase 1, but the experience was beneficial across trials in Phase 2. As R1 pointed out, our results show statically significant differences in learning and performing the new task between groups with different experience histories, which is our main claim. This demonstrates a persistent effect of prior experience across stages of learning. We have revised the text to emphasize these findings. Reviewer 2 In Figure 2C, it is unclear why all animals start with a reward in locations 2 or 3, except for the last animal, which also starts at location 3 but is not rewarded. Unlike the other examples, the last animal did not proceed to location 1. However, it is still unclear why it was not initially rewarded like the other animals. We agree with Reviewer 2 that the task rule needs to be clarified. We have updated the "Experimental design and animal training" section of the Materials and methods to explain that the reward rule. When the animal is placed in the center of the maze at each session and is free to choose the first where to go. The control program is initiated at a random step in the visit location sequence (1, 2, 1, 3). Thus, it is not always the case that the first visit made by the animal will be rewarded. This is to ensure the animals do not develop a stereotyped action strategy at the start of each session but must correctly identify the sequence. Further, in the same figure, for the first animal of the non-switching group, animals are rewarded when they visit 3-1-2-1-3-1 with some unrewarded choices in between. However, the subsequent three visits to location 1 are rewarded. Does this imply that location 1 continues to be rewarded until the next correct choice is made? The example graphs need verification, or the description of reward delivery requires clarification. This is correct. We have added clarifications to the Methods and Results. Repeating the (1, 2, 1, 3) sequence will ensure every trial is rewarded. However, when the animal is at 2 or 3, whether rewarded or not, it will be rewarded if it returns to 1. This reinforces an alternation behavior between 1 and either 2 or 3. Thus, returning to 1 is one critical step for developing the alternation. This is the same rule as previously published maze alternation tasks (Frank et al., 2000; Kim and Frank, 2009; Jadhav et al., 2012). Additionally, Figure 4 would benefit from improved legends within the figure, to make it easier to understand tau, c, alfa and beta, in particular tau. Thank you for this suggestion. We have improved the description for the model parameters in Figure 4 captions. Minor issues In Figure 2, the labels for letters F and H are not fully printed. Line 433 refers to Figure 8A, which appears to be a mistake. It should refer to Figure 6A. Line 464 refers to Figure 9A, which appears to be a mistake. It should refer to Figure 7A. We appreciate the careful read and apologize for the formatting error. These three issues have been corrected. Manuscript Instructions - Extended Data should be labeled as Figure 1-1, Figure 1-2, Table 1-1, etc., so they indicate which figure they are supporting (i.e. Extended Data table supporting Figure 5 labeled as Figure 5-1). This change is not necessary at this stage, but will be if your paper receives a revise decision. These figure references have been updated in the text
