# Supplementary figures and images for: Persistent Impact of Prior Experience on Spatial Learning
Source: eNeuro. 2024 Sep 20;11(9):ENEURO.0266-24.2024. doi: 10.1523/ENEURO.0266-24.2024 (PMC11419697; doi:10.1523/ENEURO.0266-24.2024)

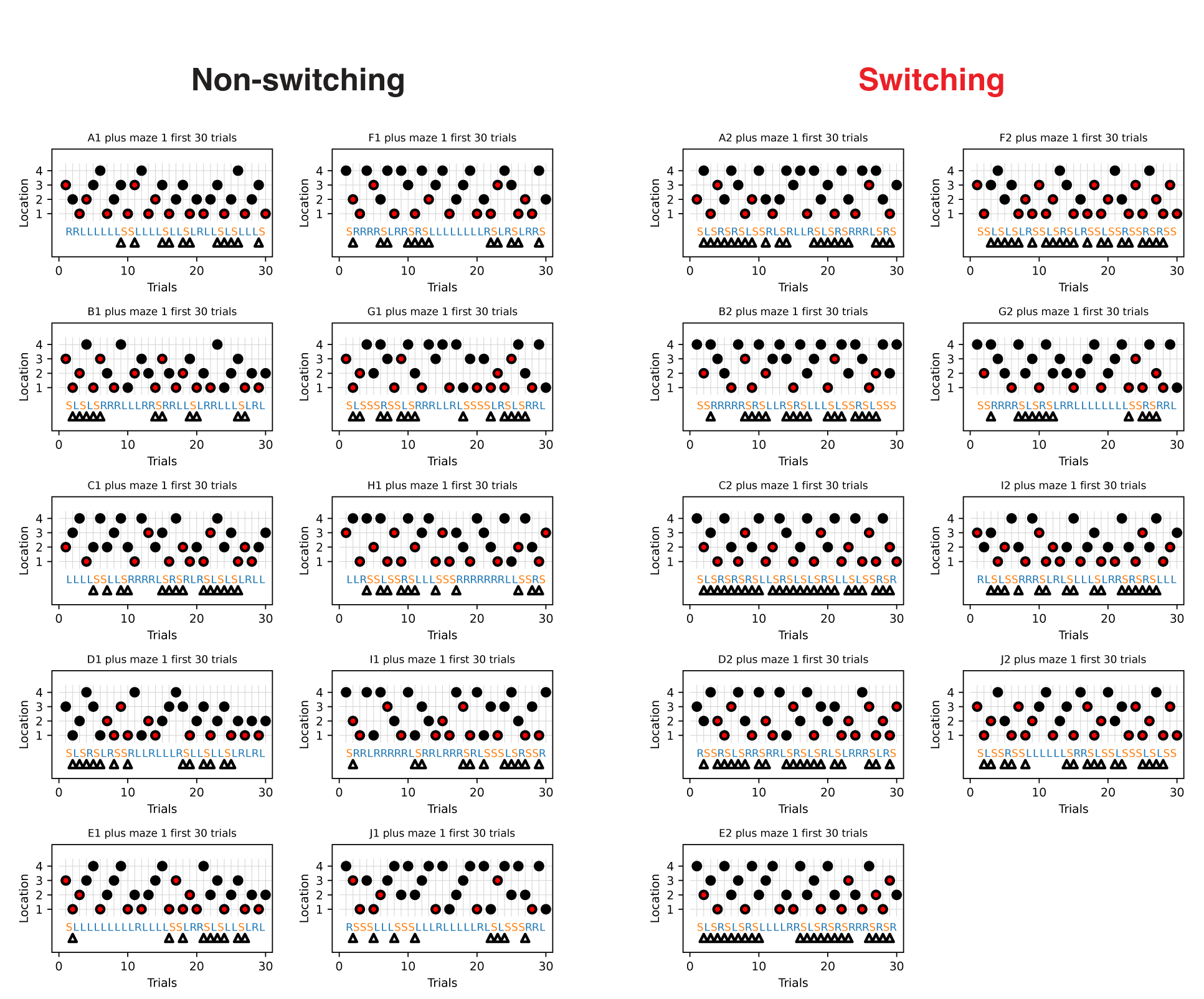

Supplement: Figure 2-1 — Behavior choices for the first 30 trials of the Plus maze for all animals, shown in the same format as Fig. 2 C-D. 1st order transitions shown by the circles that indicate the maze location visited by the rat. Red circles indicate the rewarded visits. 2nd order transitions convert the location visit pairs into left turns (L), right turns (R) and straight (S). L and R are marked blue, and S is in orange. Triangles correspond to switch trials, or 3rd order transitions that involve changes between L/R and S. Download Figure 2-1, TIF file. [file eneuro-11-ENEURO.0266-24.2024-s001.tif]

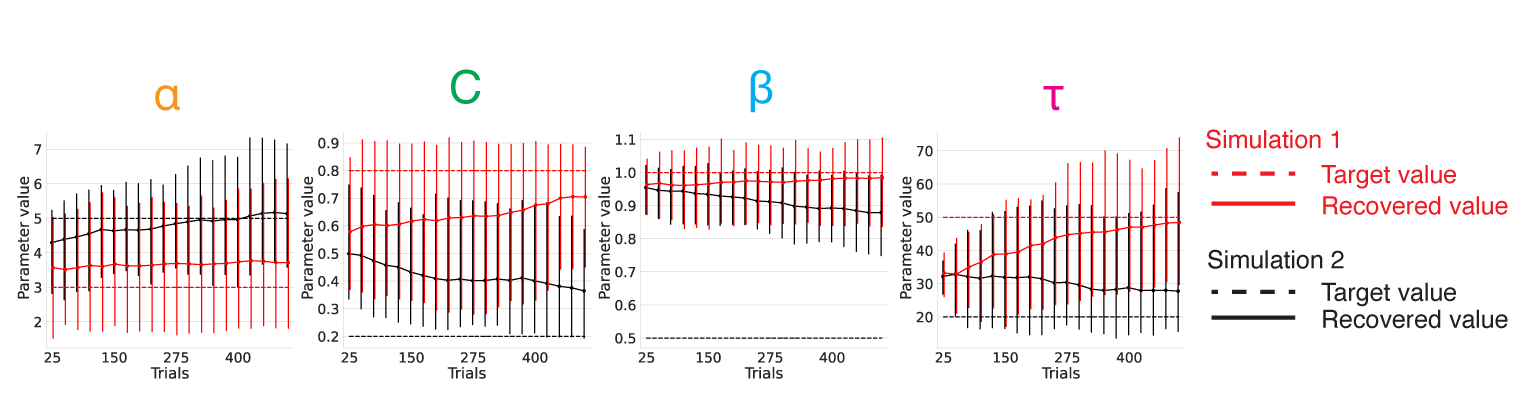

Supplement: Figure 4-1 — Simulations confirm that the model parameters can be recovered from sequences of actions. We simulated from the distance-dependent Chinese restaurant process using two different sets of parameters (simulations 1 and 2, dashed lines indicate the true parameters). For each set of parameters, we generated 50 independent simulations. The parameters were then fit with an increasing number of trials using the posterior median as the estimate. The points give the mean estimates, and the error bars show a 90% interval over simulations. The estimated parameters remained close to the prior distribution with few trials and tended towards the true parameters with increasing amounts of data. We found that the context dependency parameter (C) required the fewest number of trials to separate across these two simulations. Given low values of the chosen base distribution bias (ɑ), which meant the base distribution was unlikely to be chosen in the generated sequences compared with the history-dependent distributions, we did not expect the repetition bias parameter (β) to be effectively recovered. Download Figure 4-1, TIF file. [file eneuro-11-ENEURO.0266-24.2024-s002.tif]

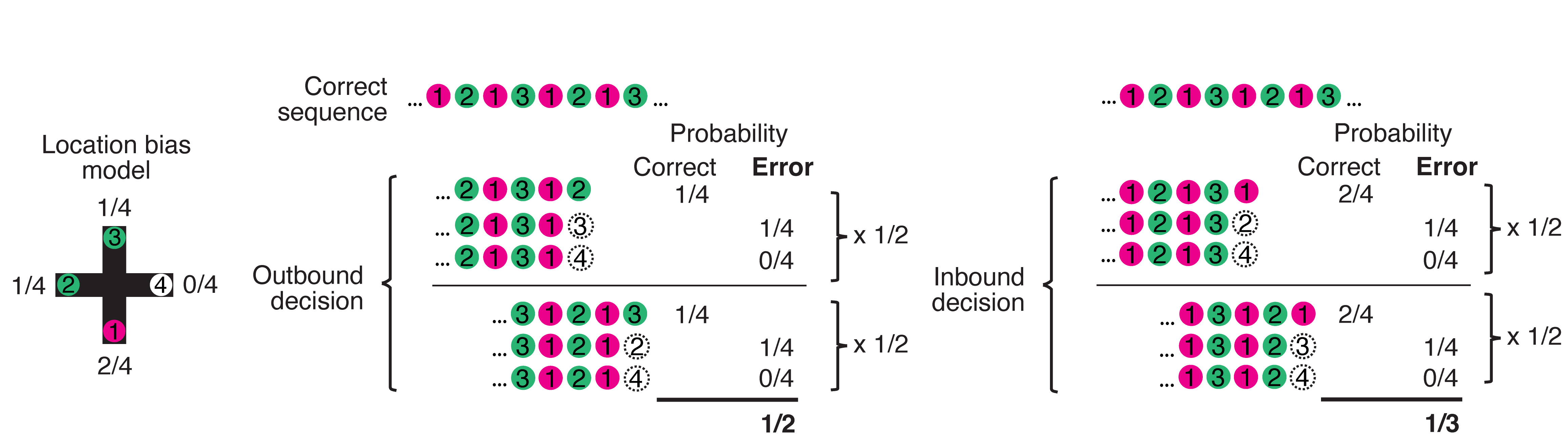

Supplement: Figure 7-2 — Error likelihood according to a location-bias strategy. Download Figure 7-2, TIF file. [file eneuro-11-ENEURO.0266-24.2024-s004.tif]
